# Laryngeal Mask Airway Method for Minimally Invasive Surfactant Therapy in Neonates with Pneumothorax Complicating Respiratory Distress Syndrome

**DOI:** 10.3390/children12020134

**Published:** 2025-01-26

**Authors:** Joaquim M. B. Pinheiro, Marilyn Fisher, Kate A. Tauber, Chad Pezzano

**Affiliations:** 1 Division of Neonatology, Department of Pediatrics, Albany Medical College, Albany, NY 12208, USA; fisherm@amc.edu (M.F.); tauberk@amc.edu (K.A.T.); pezzanc@amc.edu (C.P.); 2 Departments of Pediatrics and Cardiorespiratory Services, Albany Medical Center, Albany, NY 12208, USA

**Keywords:** neonates, pneumothorax, respiratory distress syndrome, surfactant therapy, laryngeal mask airway, supraglottic airway devices

## Abstract

Background/Objectives: Pneumothorax is a common complication of neonatal respiratory distress syndrome, which is decreased by surfactant therapy. Rescue administration of surfactant in neonates with severe RDS complicated by pneumothorax requires management of the pneumothorax to optimize surfactant distribution while avoiding positive pressure ventilation to minimize iatrogenic exacerbation of the air leak. Methods: We retrospectively reviewed our center’s experience with neonates who had clinically significant pneumothorax complicating RDS, in whom we used a novel technique to administer surfactant through a laryngeal mask/supraglottic airway device without applying positive pressure ventilation. Results: In 13 of the 20 neonates in our cohort, subsequent invasive ventilation and chest tube insertion were avoided. There were no major complications or unfavorable outcomes. We describe our experience with this method and suggest an approach to individualize the management of neonates with pneumothorax preceding surfactant therapy. Conclusions: In our setting, laryngeal mask airway devices are now the preferred method to deliver surfactant in neonates with RDS and pre-existing pneumothorax. We believe this approach is readily applicable in most neonatal care settings.

## 1. Introduction

Recent trends in the management of neonatal respiratory distress syndrome (RDS) have increasingly favored non-invasive respiratory support while avoiding intubation and invasive ventilation to deliver surfactant or assist ventilation [1]. Pneumothorax (PTX) is a common complication of neonatal RDS [2,3], with an increased incidence when surfactant therapy is delayed [4]. Rescue administration of surfactant to neonates with severe RDS complicated by PTX is traditionally accompanied by positive pressure ventilation, which may exacerbate the PTX [5]; in turn, this may trigger the need for drainage of the PTX [6,7] and/or sustained invasive mechanical ventilation, which is needed in 30–89% of cases [2]. In more severe cases, PTX may contribute to mortality, even in moderately preterm neonates [8,9].

Isolated reports of surfactant therapy to avoid tube thoracostomy in neonates with PTX have described endotracheal intubation for surfactant administration [10]. While surfactant administration continues to evolve over the last decade towards less invasive methods, with the primary purpose of minimizing exposure to positive pressure ventilation, in the mid-2010s, most neonates at Albany Medical Center still received surfactant via an endotracheal tube (ETT), either with continued invasive ventilation or as part of an Intubation–Surfactant–Rapid Extubation (INSURE) strategy. Both of these approaches involve positive pressure-assisted surfactant delivery. In that era, clinical trials assessing the comparative effectiveness of INSURE versus surfactant administration through a laryngeal mask airway device (LMA) also included positive pressure in the LMA arm [11,12]. However, these trials specifically excluded neonates with pulmonary air leaks from enrollment. As experience accrued with surfactant administration through laryngeal or supraglottic airways (recently labeled SALSA) [13], we reasoned that modifying this approach to avoid positive pressure ventilation (PPV) during surfactant delivery might be particularly desirable in neonates with a pre-existing PTX. This may avoid iatrogenic exacerbation of the PTX by PPV, while also avoiding laryngoscopy. Over the last decade, the authors’ clinical practice has gradually developed a preference for using this modified SALSA approach to administer rescue surfactant to neonates with RDS and a PTX. However, we had not assessed whether the associated outcomes are as safe and effective as traditional approaches, nor have we found any published reports on this technique.

In this study, we aimed to review our recent experience with using LMA for rescue surfactant administration without PPV in neonates with RDS complicated by PTX to estimate whether the frequency of major complications in our population compares favorably with published data. We hypothesize that this minimally invasive, individualized approach to the management of neonates with RDS and PTX is associated with a decreased need for subsequent PTX drainage and ventilation relative to those in the published literature. Such findings would support our preference for this method of surfactant delivery to neonates with RDS and pre-existing PTX; they may also reveal opportunities for further refinement of the technique.

## 2. Materials and Methods

This was a retrospective review of records on neonates admitted to the neonatal intensive care unit (NICU) at Albany Medical Center with RDS that became complicated by PTX who subsequently received rescue surfactant therapy via a laryngeal mask/supraglottic airway device. Our setting is a 60-bed level IV NICU in the regional perinatal center for northeastern New York State, admitting both inborn and transferred neonates. We screened NICU datasets containing clinical and quality improvement information for neonates admitted from 1 January 2014 to 20 December 2024 who had a diagnosis of RDS and PTX and received rescue surfactant therapy through an LMA. Neonates were excluded if the review of the medical records and chest radiograph images revealed that surfactant rescue was not performed via LMA or that PTX occurred after surfactant administration. This study was determined by the Institutional Review Board of Albany Medical Health System to be exempt research (protocol #7134, 19 December 2024), and a waiver for HIPAA authorization was approved.

We abstracted data on the baseline characteristics of the neonates and on the respiratory interventions and potential sequelae of PTX. Because of a change in electronic medical record systems in the past year, detailed physiological data were not available on some neonates. Furthermore, PTX is often associated with acute deterioration, and FiO_2_ could be rapidly increased to between 50% and 100% and adjusted multiple times while an infant was unstable, without this being recorded reliably on the medical record. Therefore, we chose not to abstract information of questionable value on FiO_2_ just before surfactant administration; however, our typical practice is to administer a first dose of rescue surfactant to neonates with surfactant deficiency or dysfunction when FiO_2_ is between 30% and 40% while on continuous positive airway pressure or non-invasive positive pressure ventilation (CPAP/NIPPV).

**Figure 1:**
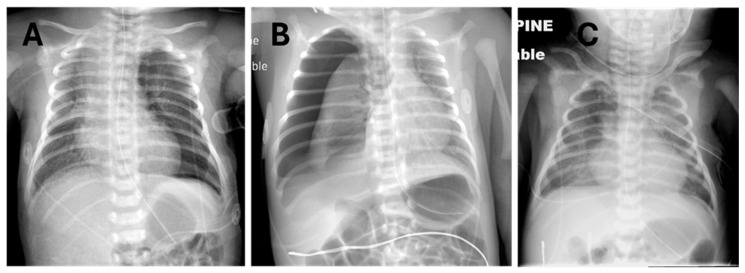
Prototypical examples of pneumothorax on chest radiographs before surfactant administration via LMA. (**A**) Left pneumothorax not drained. (**B**) Right pneumothorax prior to thoracentesis. (**C**) Left pneumothorax after chest tube drainage with residual bilateral pneumothorax and pneumomediastinum plus emphysema in the neck tissue planes.

The clinical decision on how to administer surfactant to neonates with RDS and PTX was made by the attending neonatologist, considering the individual circumstances of each patient. Briefly, it must first be decided whether or not to evacuate the pneumothorax to minimize cardiorespiratory dysfunction and optimize lung inflation to prevent maldistribution of surfactant. Examples where non-evacuation and evacuation were chosen are shown in Figure 1, panels A and B, respectively. Figure 1C exemplifies a neonate who had severe pneumothoraces and pneumomediastinum, a prior dose of surfactant, and successful evacuation of most of the pleural air with thoracentesis and a chest tube but who still had an active air leak and significant oxygen requirement yet had never been mechanically ventilated. In most instances where thoracentesis is performed, the adequacy of the procedure is assessed clinically based on the volume of gas aspirated, transillumination when feasible [14], auscultation, and acute increase in O_2_ saturations by pulse oximetry; surfactant administration is usually performed immediately after thoracentesis, without repeating a chest X-ray.

For surfactant administration, three different types of laryngeal mask/supraglottic airway (LMA/SGA) devices were available to us during this timeframe, sometimes concurrently, and the procedure notes typically do not specify the device brand. Although here we refer to the devices generically as “LMA”, in these neonates, we used size 1 models of either the LMA Unique^®^ (LMA North America, San Diego, CA, USA), i-gel^®^ (lntersurgical, Wokingham, UK), or Ambu AuraStraight^®^ (Ambu Inc., Glen Burnie, MD, USA), depending on availability and clinician preference.

Surfactants used were either poractant (Chiesi Farmaceutici, Parma, Italy) or calfactant (ONY Biotech, Amherst, NY, USA) at doses recommended by their respective manufacturers.

The technique for surfactant administration without PPV is shown in a video provided with parental permission as Supplementary Information, with identifiers removed, in a neonate with RDS whose left-sided pneumothorax had just been evacuated via catheter thoracentesis (Supplemental online Video S1). Details of the procedure as used with PPV in clinical trials have been previously published [12]. Briefly, after atropine premedication, the LMA device is inserted and held into position, and its unobstructed continuity with the glottic airway is verified in the spontaneously breathing neonate with a colorimetric CO_2_ detector. Using a short catheter (in our case, a T-connector with the distal adapter cut off), the surfactant is slowly administered into the distal half of the LMA device, allowing the newborn to aspirate the liquid while providing their own negative pressure ventilation. We deliver supplemental oxygen at the LMA connector, but we typically do not use CPAP during this brief disconnection since we are not interfering with the spontaneous expiratory closure of the vocal cords, thus allowing the infant to maintain their endogenously generated end-expiratory pressure. We avoid using PPV if possible; however, if needed, CPAP and/or a few breaths at the lowest positive pressure that promotes clearance of surfactant remaining in the LMA lumen may be briefly administered from a T-piece or anesthesia bag. After surfactant delivery, the LMA is removed and the infant returns to their previous CPAP/NIPPV support. It should be noted that in our setting, dozens of neonatal clinicians and trainees have performed the SALSA procedure, which requires minimal experience [12].

Data were collected in spreadsheet format and analyzed to generate simple descriptive statistics using Stata 18 (StataCorp, College Station, TX, USA).

## 3. Results

### 3.1. Study Population

Among the 8835 NICU admissions during the study period, 349 neonates (3.9%) were diagnosed with pneumothorax. Surfactant therapy was administered at some time to 94% (*n* = 64) of VLBW neonates with an eventual diagnosis of pneumothorax and to 35% (*n* = 97) of neonates with birthweight >1500 g with the same diagnosis. During the time frame of this study, 185 neonates were listed in the laryngeal mask implementation log as having received surfactant outside of clinical trials. Combining these two sets, we found 20 neonates with pre-existing pneumothorax who received surfactant via the LMA/SGA device, which we reviewed in more detail. One additional neonate was excluded from all analyses because partially missing records precluded ascertainment of whether the PTX preceded or followed surfactant administration.

#### Study Cohort Characteristics

The characteristics of the cohort studied are shown in Table 1.

The 20 neonates were predominantly (65%) born at term gestation, but 25% were late preterm. The majority were male and delivered by cesarean section. Their delivery room stabilization required relatively minor interventions, and none needed intubation or intensive resuscitation; the Apgar scores were mostly high. All of these neonates had an underlying pulmonary pathology, either respiratory distress syndrome due to surfactant deficiency (85% of cases), surfactant dysfunction due to meconium aspiration (10%), or blood aspiration (5%); there were no cases of transient tachypnea of the newborn (TTN). All infants were receiving nasal CPAP or NIPPV prior to rescue surfactant therapy via LMA. One infant had previously been mechanically ventilated and received surfactant via ETT; another had previously received a dose of surfactant via a thin catheter. PTX evacuation by thoracentesis before rescue surfactant therapy was required in 50% of the neonates, whose pneumothoraces were deemed large; one neonate had a chest tube before surfactant via LMA. In this population, the significant pneumothoraces developed relatively late as the severity of RDS increased, such that rescue surfactant was administered at a median of 23.3 h of age.

### 3.2. Study Outcomes

Notable outcomes in this cohort are shown in Table 2.

Following the administration of surfactant via LMA, 65% of the neonates did not require any additional intervention to treat the pneumothorax, whereas 20% needed a first thoracentesis and 15% a repeat thoracentesis. Ultimately, a chest tube was inserted for persistent pneumothorax in 30% of neonates in our cohort; concurrently, to support persistent pulmonary dysfunction and/or need for significant analgosedation owing to tube thoracostomy, 25% of neonates received conventional or high-frequency invasive mechanical ventilation; no neonate in this cohort received inhaled nitric oxide therapy. The acute pulmonary disease resolved relatively quickly, requiring a median of 5 days of supplemental oxygen. Only one preterm neonate still needed oxygen at 28 days of age, but none had chronic lung disease or required oxygen at discharge. All neonates in this cohort survived, with a median length of NICU stay of 11 days.

## 4. Discussion

In this study, we demonstrate the feasibility of a modified approach for administering rescue surfactant via LMA to a specific subset of neonates with respiratory distress syndrome complicated by pneumothorax, achieving satisfactory results while using less invasive interventions. The key modification of the SALSA technique [13] we had used in our prior clinical trials [11,12] involves avoidance of PPV when delivering surfactant via the airway device.

We note that this small cohort is not necessarily representative of the larger population of neonates with RDS and PTX since the patients were treated primarily contingent on their size, which permitted the use of a size 1 LMA; in addition, these infants had a PTX before surfactant therapy. Consequently, two-thirds of the cohort were born at term, while the majority of term neonates with pneumothorax in our setting did not require surfactant; conversely, the fact that all these neonates needed surfactant therapy may indicate a subset with greater severity of illness than the typical late preterm or term newborn with pneumothorax. Furthermore, all neonates in this cohort had underlying lung disease (by radiologist’s interpretation, confirmed by a review of all X-rays by J.M.B.P. and K.A.T.), which increases the risk of complications and mortality in neonatal PTX [15]. Because of the high likelihood of selection bias in our cohort, we will minimize potentially inappropriate comparisons with other subgroups in our setting or with published data.

The incidence of PTX decreases as gestational maturity increases [9], but the likelihood of RDS and PTX and the need for surfactant therapy are markedly increased in late preterm and early-term neonates in comparison with those born at full term [16]. In this more mature population, pneumothorax can occur spontaneously, without apparent underlying disease [17], or in association with the use of CPAP during delivery room resuscitation [18]. More commonly, it is associated with some underlying lung pathology [17], including RDS and aspiration syndromes, which can cause surfactant dysfunction and respond to surfactant therapy [19]. In our cohort, 85% of neonates had primary surfactant deficiency (RDS), and the rest had aspiration syndromes—none had TTN or spontaneous PTX. Concordant with our cohort’s characteristics, the risk of pneumothorax has been reported to be higher in male infants and those born by cesarean section [20].

Interventions to drain the PTX are not required in all cases. In the population underlying a randomized trial of neonatal PTX drainage, approximately 40% of PTXs were managed without drainage [7]. This is similar to our cohort, in which half of the pneumothoraces did not undergo drainage prior to surfactant administration. When PTX drainage is required, catheter or needle aspiration is preferable as a first step, as it reduces the need for chest tubes or drains by about 50%, and it also decreases the need for mechanical ventilation in neonates >32 weeks’ gestation [7].

In our cohort, most neonates avoided endotracheal ventilation, whereas 25% required invasive ventilation. This compares favorably with a large study of neonates with PTX, where 30% of those weighing >2500 g and 89% of those <2500 g received mechanical ventilation [2].

**Figure 2:**
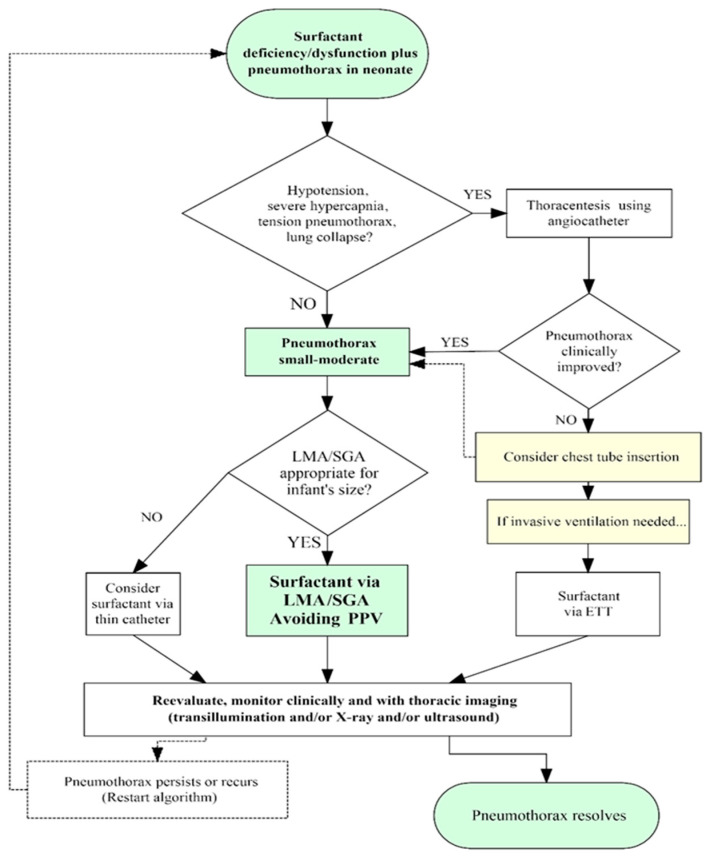
Flow diagram depicting our current approach to surfactant therapy in neonates with surfactant-related disorders and pre-existing pneumothorax.

Our approach to PTX in neonates with underlying deficiency and/or dysfunction of pulmonary surfactant is sketched in Figure 2. A gradual deterioration with increasing FiO_2_ requirement and thoracic imaging revealing PTX and/or pneumomediastinum without major lung collapse suggests a small-to-moderate PTX, which can usually be managed by prompt surfactant administration by LMA, avoiding PPV, and without the need for thoracentesis. The findings that are indicative of a large PTX that requires evacuation through thoracentesis before surfactant administration include acute clinical deterioration, cardiorespiratory instability, and a chest radiograph showing tension PTX and/or lung collapse. Initial intervention should be prompt, aiming to render the PTX small–moderate in order to administer surfactant safely and homogeneously but without delay. Indicators of successful thoracentesis include the removal of a significant volume of pleural gas (often 20–30 mL or greater), the immediate increase in pulse oximetry saturation readings, and especially an obvious decrease in PTX size by transillumination (when conditions are favorable for using this method). Repeating a chest radiograph at this stage is not recommended since it introduces undesirable delays; furthermore, the single delayed image may miss a transiently successful thoracentesis, which might have permitted surfactant administration before the PTX reaccumulated.

In neonates too small for a size 1 LMA (in our setting, below 1000–1200 g birth weight, depending on available device and user expertise), surfactant can be administered via a thin catheter. Thin catheter methods avoid PPV, but they require laryngoscopy and entail a higher degree of technical difficulty and clinician expertise. In the less common cases where thoracentesis fails to ameliorate the PTX, a chest tube or pleural drain must be considered. In such situations, respiratory failure may prompt the need for intubation and invasive ventilatory support (which may also exacerbate the air leak). The sequence of interventions in such cases must be individualized. Nevertheless, the immediate goals are to stabilize ventilation, allow reinflation of the collapsed lung(s), deliver surfactant promptly and homogeneously, and ideally return the patient to CPAP/NIPPV as soon as possible.

Irrespective of the initial severity of the PTX and the method of surfactant administration, subsequent monitoring and reassessment should account for the evolution of both the PTX and the underlying surfactant deficiency, which do not necessarily resolve in tandem. Some neonates respond to surfactant administration by immediately weaning off supplemental O_2_, yet their PTX may resolve slowly over a few days. In others, the PTX may be immediately evacuated and not recur, but they may require re-dosing of surfactant over the next day or two. Combinations of these features require an individualized approach to monitoring and treating each neonate. In neonates with air leaks, we favor a lower threshold for using surfactant rather than escalating non-invasive or invasive ventilatory support pressures. For example, the infant in Figure 1C had received an earlier dose of surfactant and eventually required a chest tube to control the PTX, pneumomediastinum, and neck emphysema. Although the PTX was relatively insignificant on that chest X-ray, the inability to wean FiO_2_ prompted another dose of surfactant via LMA, to which the baby responded well, allowing for the removal of the chest tube, weaning off respiratory support, and discharge to home without symptoms or the need for further X-rays. As with therapeutic interventions for the RDS and PTX, diagnostic monitoring also needs to be customized for individual infants and their care settings. We use post-thoracentesis X-rays selectively since other options for structural and functional monitoring of respiratory function in RDS and pneumothorax are available. Post-evacuation X-rays involve radiation exposure and cannot track rapidly changing lung conditions in real time. Imaging with transillumination [14] or lung ultrasound [17] permits real-time monitoring of PTX without radiation exposure. Transillumination allows evaluation of the approximate size and location of the PTX, but it is less reliable in large newborns, particularly when environmental darkness cannot be achieved [14]. Point-of-care lung ultrasound has recently been used by specifically trained clinicians to detect neonatal PTX that require evacuation, with accuracy comparable to X-ray and transillumination [17,21]; however, it is uncertain whether it can reliably quantify the size of the PTX, particularly after thoracentesis, and few clinicians have expertise in this method. We suggest that imaging by one or more modalities is most useful in the rapid diagnosis of PTX and to guide evacuation if needed; subsequently, clinical monitoring, especially continuous pulse oximetry, is most useful, and it may be sufficient if clinical evidence of respiratory dysfunction resolves.

Our observations are limited by the descriptive nature of this study, precluding direct comparisons with alternate approaches, either in our NICU or in published data. Furthermore, our approach is not strictly standardized, having evolved over the last few years, particularly as we have lowered the clinical threshold to employ this procedure. It is likely that our ability to predict the need for surfactant therapy will improve over time, and that the population benefiting from this approach will change, in our NICU. The applicability of this technique to other NICU settings will depend on the approach to the management of RDS, the frequency of PTX in various subgroups of neonates, the availability of LMA/SGA devices of various sizes, and the experience of clinicians in using them. Currently available LMAs are generally recommended for use in newborns weighing >1500 g, although the same devices have been used by experts in newborns weighing 1200 g [22] and even down to 1000 g [12]. It must be noted that while surfactant therapy is a standard in neonatal care, there is no FDA-approved device specifically to administer liquid surfactant; endotracheal tubes, vascular and feeding catheters, and laryngeal mask devices have all been used off label by clinicians for this purpose. Nevertheless, our report demonstrates the feasibility of using an LMA/SGA device as a simple option for administering surfactant to neonates with PTX-complicating RDS or surfactant dysfunction disorders, which could easily be adopted in both intensive care settings and in related scenarios, such as referring hospitals and for stabilization before neonatal transports. It is possible that delivering surfactant via LMA without using PPV, or with minimal PPV only if needed, might also benefit neonates with RDS who do not have a PTX, but that remains unstudied; therefore, we still use PPV at the lowest effective pressures for routine SALSA in neonates with uncomplicated RDS, as tested in clinical trials [11,12].

## 5. Conclusions

Laryngeal mask airway devices allow us to deliver surfactant to neonates with RDS and pre-existing pneumothorax while avoiding laryngoscopy and PPV, enabling us to forgo major escalation of invasive drainage procedures and respiratory support in the majority of such neonates. Although this is now the preferred approach in our clinical practice setting, it should be regarded only as a reasonable option until higher-quality comparative studies can be conducted.

## Figures and Tables

**Table 1:** Characteristics of 20 neonates with pneumothorax prior to receiving rescue surfactant via the laryngeal mask airway device.

Characteristic	Frequency
Pneumothorax pre-surfactant	20 (100%)
Cesarean delivery	14 (70%)
Gestational weeks, mean (range)	36.4 (27–42)
Preterm (GA <37 weeks) Late preterm (GA 34–<37 weeks)	7 (35%) 5 (25%)
Birth weight in grams, mean (range)	2790 (1280–3865)
Male sex	14 (70%)
Non-Hispanic white race/ethnicity	20 (100%)
Inborn	10 (50%)
Delivery room resuscitation: None Oxygen CPAP Face mask positive pressure ventilation (PPV) Intubation, epinephrine, or chest compressions	8 (40%) 11 (55%) 12 (60%) 3 (15%) 0 (0%)
Apgar scores, median [interquartile range]: 1 min 5 min	8 [6.5–8.5] 8 [8–9]
Underlying pulmonary diagnosis: Respiratory distress syndrome (RDS) Transient tachypnea of the newborn (TTN) Meconium aspiration syndrome Blood aspiration	20 (100%) 17 (85%) 0 (0%) 2 (10%) 1 (5%)
Pneumothorax drainage pre-surfactant: None Thoracentesis (>1) + chest tube	10 (50%) 10 (50%) 1 (5%)
Prior surfactant therapy via ETT and/or thin catheter	2 (10%)
Hours of life at LMA surfactant rescue, mean (range)	23.3 (3.5–49.8)

Abbreviations: CPAP, continuous positive airway pressure; ETT, endotracheal tube; GA, gestational age; LMA, laryngeal mask airway.

**Table 2:** Interventions and outcomes following rescue surfactant via LMA in 20 neonates with pre-existing pneumothorax.

Intervention or Outcome	Frequency
Pneumothorax drainage post-surfactant: None Thoracentesis, initial Thoracentesis, repeat + subsequent chest tube	13 (65%) 4 (20%) 3 (15%) 6 (30%)
Respiratory support during NICU stay: CPAP NIPPV Conventional ventilation High-frequency ventilation	20 (100%) 7 (35%) 5 (25%) 2 (10%)
Supplemental oxygen days, median [interquartile range]	5 [3.5–8]
Chronic lung disease *	0 (0%)
Survived to NICU discharge: Discharged home Transferred to non-intensive care	20 (100%) 19 (95%) 1 (5%)
NICU length of stay in days, median [interquartile range]	11 (8–17)

Abbreviations: CPAP, continuous positive airway pressure; LMA, laryngeal mask airway; NICU, neonatal intensive care unit; NIPPV, nasal intermittent positive pressure ventilation. * Chronic lung disease: supplemental O_2_ at >28 days of age if GA > 31 6/7 weeks; otherwise, supplemental O_2_ at 36 weeks postmenstrual age.

## Data Availability

Study data are unavailable due to privacy concerns for this small number of patients.

## Supplementary Materials

The following supporting information can be downloaded at https://www.mdpi.com/article/10.3390/children12020134/s1, Video S1: Surfactant via LMA without PPV.mp4.

## Abbreviations

The following abbreviations are used in this manuscript:

CPAP	Continuous positive airway pressure
ETT	Endotracheal tube
GA	Gestational age
LMA/SGA	Laryngeal mask airway/supraglottic airway
NICU	Neonatal intensive care unit
NIPPV	Nasal intermittent positive pressure ventilation
PPV	Positive pressure ventilation
PTX	Pneumothorax
RDS	Respiratory distress syndrome
SALSA	Surfactant administration through laryngeal or supraglottic airway
TTN	Transient tachypnea of the newborn

## References

[1] de Carvalho Nunes G., Barbosa de Oliveira C., Zeid M., Leone M., Mardakis S., Remmer E., Boyer J., Hailu E., Altit G., Beltempo M. (2024). Early bubble CPAP protocol implementation and rates of death or severe BPD. Pediatrics.

[2] Aly H., Massaro A., Acun C., Ozen M. (2014). Pneumothorax in the newborn: Clinical presentation, risk factors and outcomes. J. Matern. Fetal Neonatal Med..

[3] Tawfik D.S., Gould J.B., Profit J. (2019). Perinatal risk factors and outcome coding in clinical and administrative databases. Pediatrics.

[4] Stevens T.P., Blennow M., Myers E.H., Soll R. (2009). Early surfactant administration with brief ventilation vs. selective surfactant and continued mechanical ventilation for preterm infants with or at risk for respiratory distress syndrome [Systematic Review]. Cochrane Database Syst. Rev..

[5] Niwas R., Nadroo A.M., Sutija V.G., Gudavalli M., Narula P. (2007). Malposition of endotracheal tube: Association with pneumothorax in ventilated neonates. Arch. Dis. Child. Fetal Neonatal Ed..

[6] Vibede L., Vibede E., Bendtsen M., Pedersen L., Ebbesen F. (2017). Neonatal pneumothorax: A descriptive regional Danish study. Neonatology.

[7] Murphy M.C., Heiring C., Doglioni N., Trevisanuto D., Blennow M., Bohlin K., Lista G., Stucchi I., O’Donnell C.P.F. (2018). Effect of needle aspiration of pneumothorax on subsequent chest drain insertion in newborns: A randomized clinical trial. JAMA Pediatr..

[8] Dargaville P.A., Gerber A., Johansson S., De Paoli A.G., Kamlin C.O.F., Orsini F., Davis P.G. (2016). Incidence and outcome of CPAP failure in preterm infants. Pediatrics.

[9] Acun C., Nusairat L., Kadri A., Nusairat A., Yeaney N., Abu Shaweesh J., Aly H. (2021). Pneumothorax prevalence and mortality per gestational age in the newborn. Pediatr. Pulmonol..

[10] Soliman Y., Babu T.A. (2018). Surfactant for tension pneumothorax in term neonates. Dodging chest tubes with a novel approach. Neonatol. Today.

[11] Pinheiro J.M.B., Santana-Rivas Q., Pezzano C. (2016). Randomized trial of laryngeal mask airway versus endotracheal intubation for surfactant delivery. J. Perinatol..

[12] Gallup J.A., Ndakor S.M., Pezzano C., Pinheiro J.M.B. (2023). Randomized trial of surfactant therapy via laryngeal mask airway versus brief tracheal intubation in neonates born preterm. J. Pediatr..

[13] Guthrie S.O., Fort P., Roberts K.D. (2021). Surfactant administration through laryngeal or supraglottic airways. NeoReviews.

[14] Wyman M.L., Kuhns L.R. (1977). Accuracy of transillumination in the recognition of pneumothorax and pneumomediastinum in the neonate. Clin. Pediatr..

[15] Esme H., Dogru O., Eren S., Korkmaz M., Solak O. (2008). The factors affecting persistent pneumothorax and mortality in neonatal pneumothorax. Turk. J. Pediatr..

[16] Hibbard J.U., Wilkins I., Sun L., Gregory K., Haberman S., Hoffman M., Kominiarek M.A., Reddy U., Bailit J., Branch D.W. (2010). Respiratory morbidity in late preterm births. JAMA.

[17] Jhaveri V., Vali P., Giusto E., Singh Y., Lakshminrusimha S. (2024). Pneumothorax in a term newborn. J. Perinatol..

[18] Smithhart W., Wyckoff M.H., Kapadia V., Jaleel M., Kakkilaya V., Brown L.S., Nelson D.B., Brion L.P. (2019). Delivery Room Continuous Positive Airway Pressure and Pneumothorax. Pediatrics.

[19] Keiser A., Bhandari V. (2016). The Role of Surfactant Therapy in Nonrespiratory Distress Syndrome Conditions in Neonates. Am. J. Perinatol..

[20] Benterud T., Sandvik L., Lindemann R. (2009). Cesarean section is associated with more frequent pneumothorax and respiratory problems in the neonate. Acta Obstet. Gynecol. Scand..

[21] Cattarossi L., Copetti R., Brusa G., Pintaldi S. (2016). Lung ultrasound diagnostic accuracy in neonatal pneumothorax. Can. Respir. J..

[22] Smee N.J., Boyd D., Conetta H., O’Shea J. (2021). Laryngeal mask airway surfactant administration: Case series of 60 infants. Arch. Dis. Child. Fetal Neonatal Ed..

